# Two-stage optical system for colorectal polyp assessments

**DOI:** 10.1007/s00464-015-4186-x

**Published:** 2015-04-04

**Authors:** Mirosław Szura, Artur Pasternak, Krzysztof Bucki, Katarzyna Urbańczyk, Andrzej Matyja

**Affiliations:** First Department, General, Oncological and Gastrointestinal Surgery, Jagiellonian University Medical College, 40th Kopernika St., 31-501 Kraków, Poland; Department of Anatomy, Jagiellonian University Medical College, 12th Kopernika St., 31-034 Kraków, Poland; MEDICINA - Specialist Diagnostic & Therapeutic Centre, 5th Rogozinskiego St., 31-559 Kraków, Poland; Department of Pathomorphology, Jagiellonian University Medical College, 16th Grzegórzecka St., 31-531 Kraków, Poland

**Keywords:** Colonoscopy, Colorectal cancer, Narrow-band imaging, Dual-focus magnification

## Abstract

**Background and aims:**

Macroscopic real-time evaluations of the histopathology and degree of invasion of colorectal polyps help to select the most suitable endoscopic treatment method. Dual-focus (DF) narrow-band imaging (NBI) is a new imaging enhancement system that uses digital and optical methods to enhance the view of blood vessels on mucosal surfaces. However, the superiority of this technique over standard imaging techniques has not been previously reported. The aim of this study was to determine whether the two-stage optical systems in a new generation of endoscopes will increase the diagnostic accuracy of colorectal polyp recognition.

**Methods:**

The study included 270 patients, and 386 colorectal polyps were diagnosed and removed. The polyps were assessed with white light and NBI using one- and two-stage optical systems, respectively. After being classified according to the Kudo pit pattern schemes, the polyps were removed and histopathologically verified.

**Results:**

Regarding non-neoplastic lesions (Kudo I and II), no difference was observed in the recognition of polyps when using the NBI-DF function. We observed improved accuracy in the preliminary diagnoses of Kudo III_L_ lesions (from 87.16 to 90.09 %, *p* < 0.05) and Kudo III_S_ lesions (from 87.29 to 92.79 %, *p* < 0.01). NBI-DF also increased the accuracy of preliminary diagnoses of Kudo IV lesions (from 88.24 to 94.12 %, *p* < 0.01). The Kudo V pit patterns were also more distinct with NBI-DF imaging, increasing the diagnostic accuracy from 91.67 to 100 %.

**Conclusions:**

Using a two-stage optical system with electronic colorization of the mucosa increased diagnostic accuracy for differentiating colorectal polyps with neoplastic potential.

Colorectal cancer (CRC) is the third most common cancer and the second leading cause of cancer deaths worldwide [1]. Most colorectal cancers stem from preexisting adenomatous polyps, following the adenoma–carcinoma sequence [2, 3]. Colonoscopy is widely used to diagnose colorectal cancer, as well as to detect and remove adenomatous polyps. Optical endoscopy has been endorsed as the preferred CRC screening strategy for CRC prevention beginning at age 50 [4, 5]. The ability to distinguish between benign and malignant lesions using endoscopy is crucial. However, endoscopy using conventional white light (WL) imaging alone is frequently insufficient for a preliminary, real-time diagnosis. Narrow-band imaging (NBI), also known as “electronic chromoendoscopy,” is a newly developed technology that provides a unique opportunity to assess surface mucosal and vascular patterns on polyps, potentially providing an in vivo histological diagnosis [6]. NBI uses optical filters for red, green, blue (RGB) sequential illumination and narrows the bandwidth of spectral transmittance [7]. This technique enables the observation of fine capillaries in the superficial mucosa of the gastrointestinal tract. NBI may have the potential to improve the detection rate of colorectal polyps compared to conventional WL colonoscopy, particularly for small and flat lesions [8]. Kudo et al. described a pit pattern classification system for colorectal neoplasia (type I to type V) [9–15]. In the colorectal field, pit pattern diagnosis is clinically significant because it can differentiate between neoplasia and non-neoplasia, diagnose the degree of histological atypia in a tumor, diagnose the invasion depths of early carcinomas, detect minute residual tumors after endoscopic resection, estimate the degree of histological inflammation in ulcerative colitis, and diagnose dysplasia-/colitis-associated carcinomas in ulcerative colitis [16]. Usually, standard magnification requires the use of chromoagents (e.g., indigo carmine, crystal violet, or methylene blue) to clarify the pit structures in these diagnostic procedures. Simpler and more convenient procedures are desirable for magnifying procedures. It has been suggested that NBI colonoscopy is as effective as chromoendoscopy in differentiating between neoplastic and non-neoplastic colorectal lesions using pit pattern classification, and its diagnostic accuracy is much greater than that of conventional WL colonoscopy.

In addition to NBI, a novel diagnostic technique has recently emerged, the so-called dual-focus (DF) magnification (Olympus Optical Co. Ltd, Tokyo, Japan). DF two-stage optical lens technology from Olympus allows physicians to switch from a normal focus mode to the near focus (NF) mode, enabling up to 100× magnification with the single push of a button. This visualization allows for the close examination of mucosal tissue and capillary networks.

The aim of this study was to evaluate and compare the diagnostic characteristics of a novel NBI system with DF magnification function in differentiating colorectal polyps.

## Materials and methods

The study was conducted at MEDICINA Specialist Diagnostic & Therapeutic Centre (a private hospital that performs approximately 6000 colonoscopies each year), in Cracow, Poland. The study was approved by the local ethics committee and was conducted in accordance with the principles of the Declaration of Helsinki. As a clinical trial, the study was registered in a centralized clinical trials registry (ClinicalTrials.gov - NCT01688557). All authors of this study had access to the study data and had reviewed and approved the final manuscript.

**Fig. 1:**
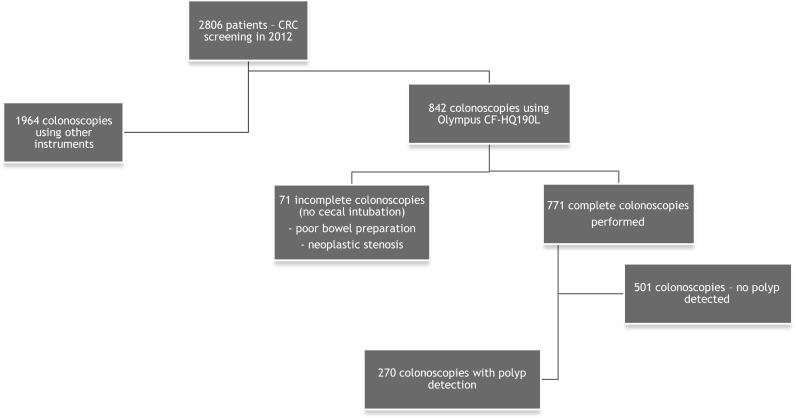
CONSORT diagram of patient enrollment

We included patients 40–65 years old who underwent the procedure in the context of an opportunistic colorectal cancer screening. In addition to age, the following exclusion criteria were applied: symptoms of colon cancer (e.g., bleeding unrelated to hemorrhoids), changes in bowel movement regularity, and unexplained weight loss. Patients who had already received a colonoscopy within the last 10 years were also excluded. A total of 2806 patients were referred and scheduled for outpatient colonoscopy in 2012 as a part of a national colorectal cancer-screening program. Of these patients, 842 were examined using a 190 series Exera III NBI system (CF-HQ190L, Olympus Co. Ltd, Tokyo, Japan) with DF capability. The cecum was successfully intubated in 771 patients, whereas the remaining 71 subjects were excluded due to inadequate bowel preparation or neoplastic infiltration occluding the lumen. Colorectal polyps were detected in 270 patients (35 %) who were prospectively enrolled in the study (Fig. 1).

All patients were given the same bowel preparation guidelines based on the oral ingestion of liquid propulsive agents (i.e., 420 g of polyethylene glycol (PEG) in 4 L of water taken in 4 doses every 6 h one day before the colonoscopy). Each colonoscopy was performed by one of the three experienced endoscopists (each of whom has performed more than 5000 colonoscopies).

**Fig. 2:**
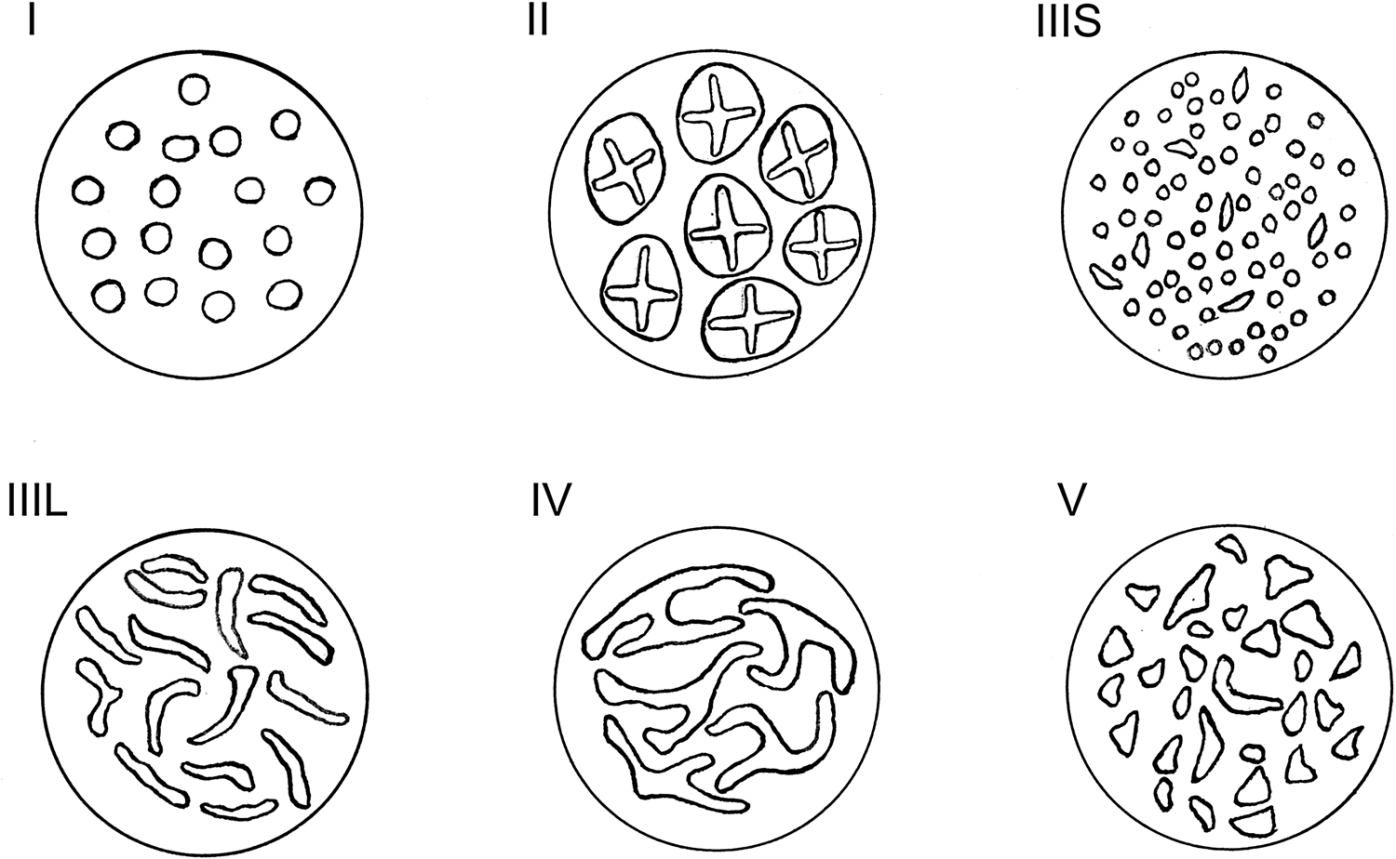
Pit pattern classification of colorectal neoplasia (Kudo et al.). *I* Round pit (normal pit), *II* asteroid pit, *III*_*S*_ tubular of round pit (smaller than the normal pit, i.e., type I), *III*_*L*_ tubular of round pit (larger than the normal pit, i.e., type I), *IV* dendritic or gyrus-like pit, *V* amorphous, nonstructured pit

**Fig. 3:**
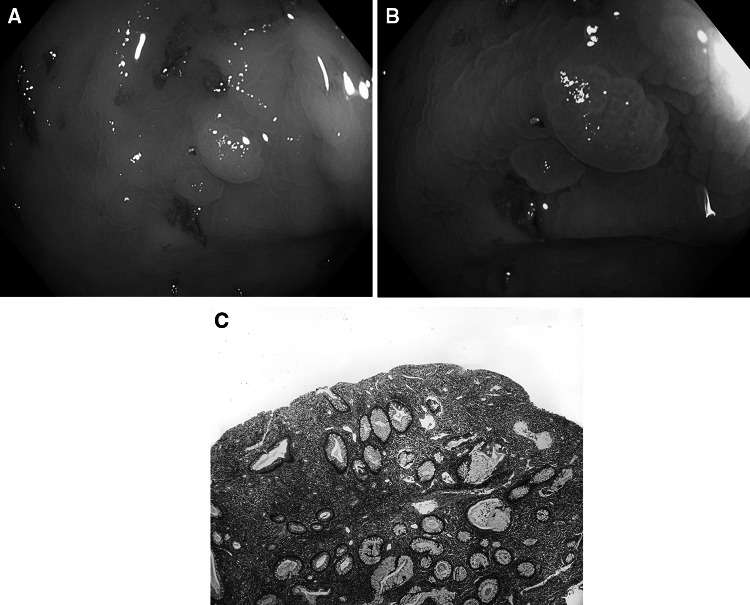
Inflammatory polyp. **A** Standard colonoscopic view in WL. **B** NBI-NF/Kudo I/. **C** Cross section (HE staining)

**Fig. 4:**
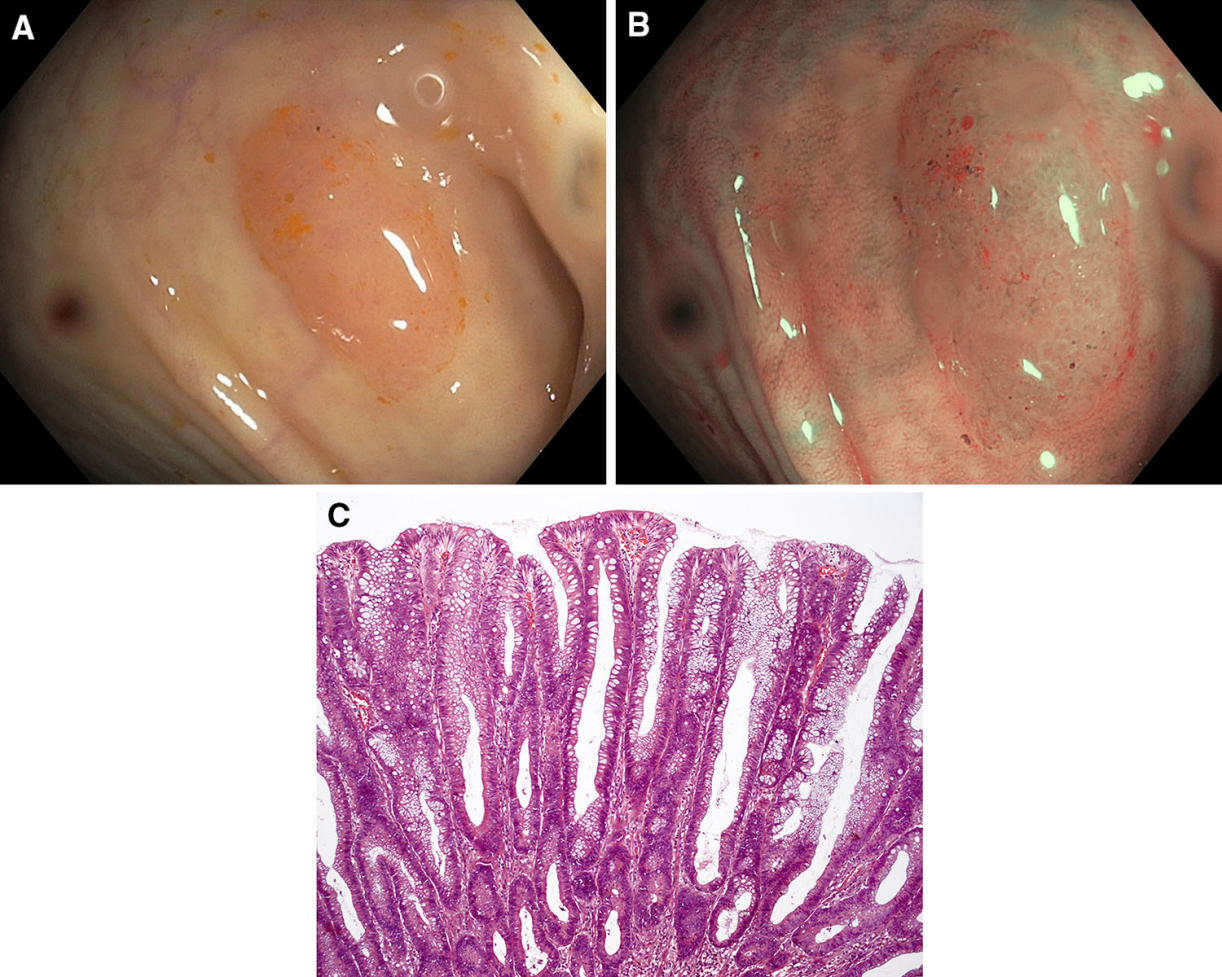
Hyperplastic polyp. **A** Standard colonoscopic view in WL. **B** NBI-NF/Kudo II/. **C** Cross section (HE staining)

**Fig. 5:**
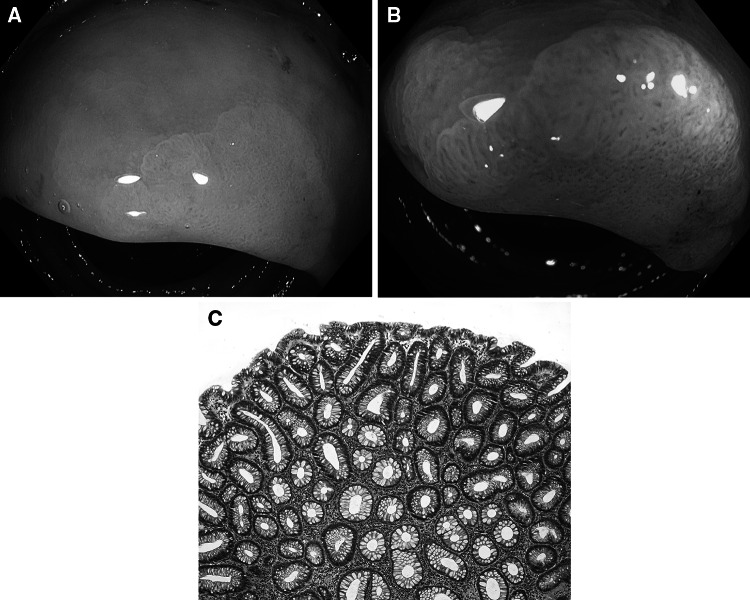
Tubular adenoma with low-grade dysplasia. **A** Standard colonoscopic view in WL. **B** NBI-NF/Kudo III_S_/. **C** Cross section (HE staining)

**Fig. 6:**
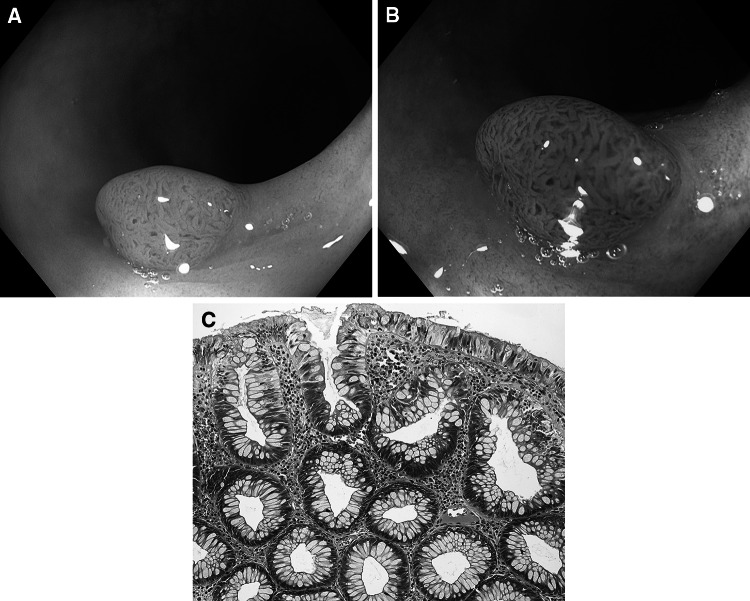
Tubular adenoma with low-grade dysplasia. **A** Standard colonoscopic view in WL. **B** NBI-NF/Kudo III_L_/. **C** Cross section (HE staining)

**Fig. 7:**
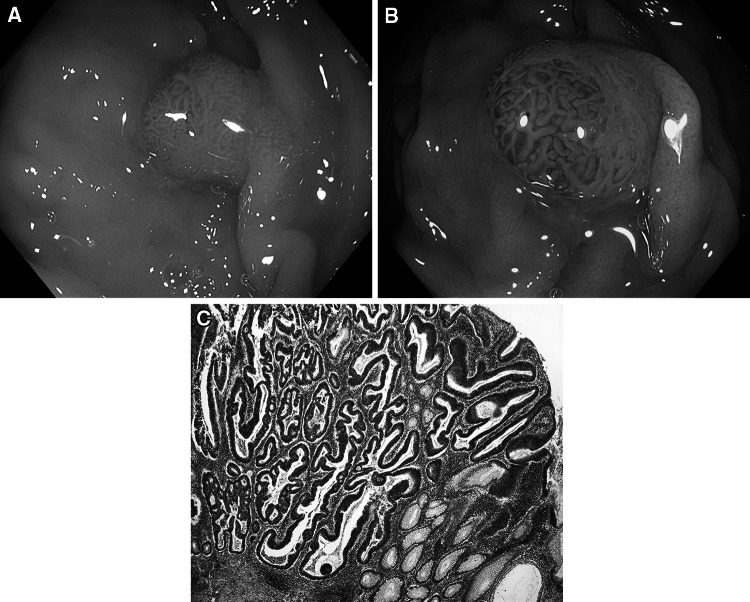
Tubular adenoma with high-grade dysplasia. **A** Standard colonoscopic view in WL. **B** NBI-NF/Kudo IV/. **C** Cross section (HE staining)

**Fig. 8:**
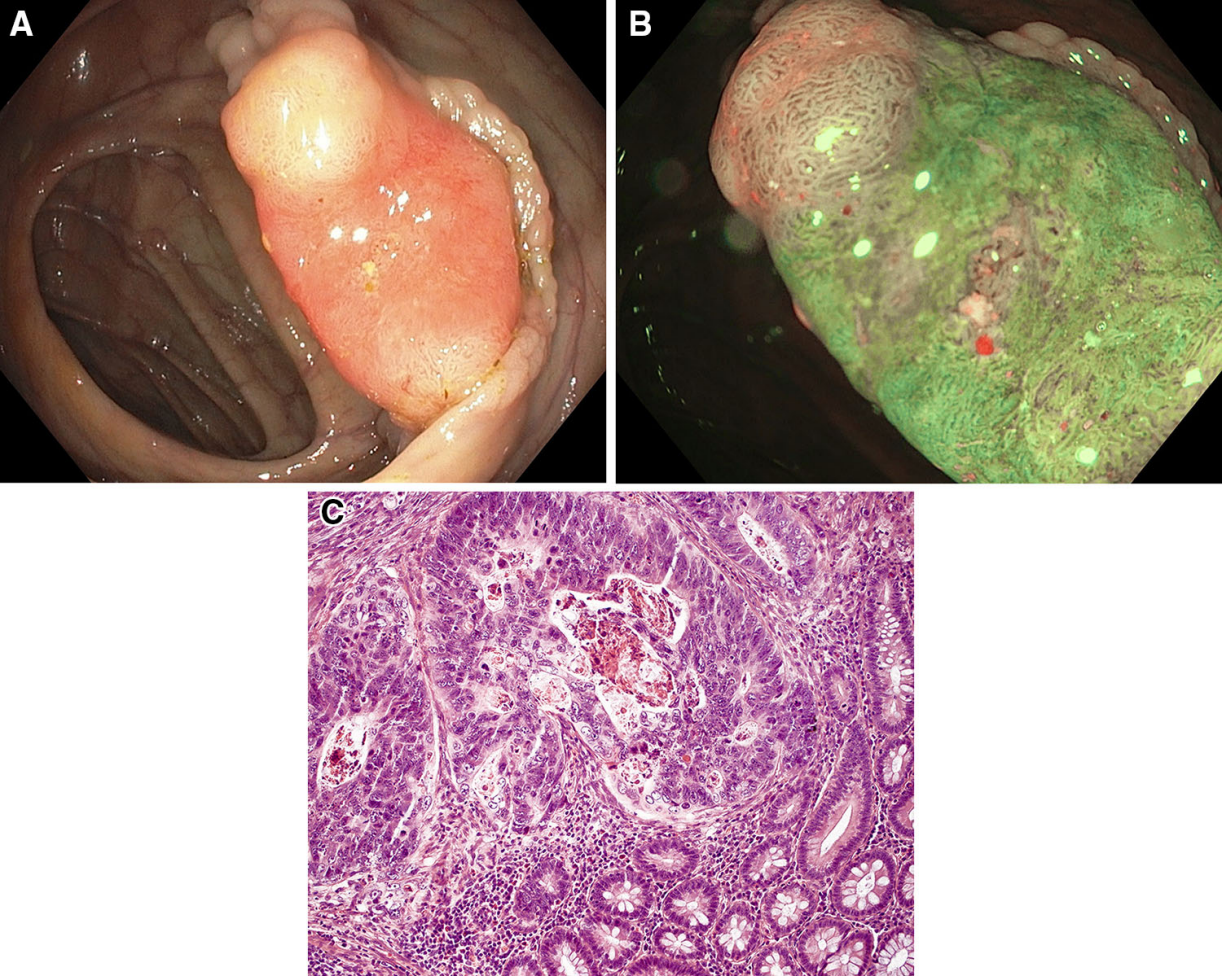
Cribriform comedo-type adenocarcinoma G3. **A** Standard colonoscopic view in WL. **B** NBI-NF/Kudo V/. **C** Cross section (HE staining)

A standard, commercially available high-definition colonoscope (190 series Exera III NBI system with DF capability) was used for all procedures in this study. Moreover, all examinations were performed with magnetic endoscope imaging (ScopeGuide, Olympus Optical Co. Ltd, Tokyo, Japan), which helped to avoid loop formation upon insertion and improved the accuracy of anatomical localization and endoscope positioning. These changes were crucial not only for conducting an effective procedure but also for localizing the pathological lesions more precisely within the large intestine. After cecal intubation, the colonic mucosa was carefully visualized with WL while withdrawing the colonoscope. All polyps detected during the procedure were documented for size, location, and morphology (i.e., pedunculated, sessile, or flat). Each polyp was routinely evaluated in real time, initially with WL and later with NBI and NBI-DF. All images were captured and stored as high-definition JPEG files (200–300 kb, 1280 × 1024 pixel array, 32-bit RGB representation). Histology was predicted for all polyps in vivo using WL, NBI, and NBI-DF based on the surface mucosal and vascular patterns identified by the respective techniques. Following these evaluations, the lesions were classified according to Kudo’s pit pattern classification and subsequently resected endoscopically or surgically. Kudo proposed a gross classification of pit patterns into seven types. It has been suggested that type I and II pit patterns are characteristic of non-neoplastic lesions, such as normal mucosa or hyperplastic polyps. However, most lesions showing pattern types III_S_, III_L_, or IV, as well as a subset of V_I_, are intramucosal neoplastic lesions (e.g., adenoma or intramucosal carcinoma). Lesions with a type V_N_ pattern and a subset of type V_I_ are suggestive of deep invasive carcinoma (Fig. 2) [17, 18]. Thus, type I and II lesions were designated as non-neoplastic patterns, and all other types were neoplastic. All polyps detected during the examination were removed after the endoscope was withdrawn. Polyps with a diameter of < 3 mm were resected using biopsy forceps, without diathermy; polyps measuring 4–7 mm were resected by endoscopic loop, without diathermy; and the larger lesions were removed by endoscopic loop, with diathermy, or endoscopic mucosal resection. Resected specimens were stained with hematoxylin and eosin (HE) and reviewed by an expert gastrointestinal pathologist with more than 10 years of gastrointestinal pathology experience. The pathologist was blinded to the endoscopic findings. The WL, NBI, and NBI-DF predictions were then compared with the final histopathological diagnosis (Figs. 3, 4, 5, 6, 7, 8).

### Statistics

The materials acquired in this study were systematized and analyzed, and a distribution of the variables was established. Because the analyzed parameters do not have a normal distribution, nonparametric tests were used in the analysis. Qualitative variables were compared using the independent χ^2^ test. The Mann–Whitney *U* test was used to compare quantitative variables between two groups. The Kruskal–Wallis test was used for comparisons of quantitative data in more than two groups. The materiality threshold was established at *p* ≤ 0.05.

## Results

A total of 270 patients [128 males (47.41 %) and 142 females (52.59 %); mean age 55.04 years, ±standard deviation (SD) 4.06 years; range 40–65 years] were recruited. The number of patients in each age group is summarized in Table 1. The mean body mass index (BMI) was 27.69 ± SD 4.06. The cecum was successfully intubated in all patients (100 %), and the total time required to reach the cecum was 55–470 s (mean 234 s). The withdrawal time was >6 min in each case. Patients had 1 to 5 polyps detected (mean 1.43 ± SD 0.75) (Table 2). A total of 386 polyps were detected and further analyzed macroscopically. The anatomical localization of the polyps was determined based on the endoscopic image and location presented on a ScopeGuide navigation system display. Among the analyzed patients, the polyp detection rate in particular locations of the large intestine was similar in males and females. The most frequent polyp locations were the sigmoid colon and sigmorectal junction [177 lesions (45.85 %) in total]. The distribution of the remaining lesions was as follows: rectum, 86 (22.28 %); descending colon and splenic flexure, 34 (8.81 %); transverse colon, 26 (6.74 %); ascending colon and hepatic flexure, 51 (13.21 %); and cecum, 12 (3.11 %) (Table 3). The majority of detected lesions were small polyps with a diameter measuring <10 mm (87.82 %). The morphological shape of the polyps was evaluated using the Paris classification [19]. A total of 143 polyps (37 %) were pedunculated or subpedunculated (Paris types Ip and Ips, respectively); 188 (49 %) polyps were sessile (Paris type Is); and the remaining 55 polyps (14 %) were superficial and elevated (Paris types IIa, b, c) (Table 4). The most numerous group consisted of sessile lesions with diameters up to 6 mm (Paris type Is), representing 44 % of the total number of lesions (170) (Table 4). All detected polyps were removed and subsequently evaluated histopathologically. Final histology showed that 142 polyps (36.79 %) were hyperplastic; 214 polyps were low-grade adenomas (55.44 %); 19 were high-grade adenomas (4.92 %); and 11 were adenocarcinomas (2.85 %) (Table 5). The histopathological distribution of polyps in terms of their macroscopic appearance is shown in Table 5.

**Table 1:** Number of patients by age groups

Sex	Age groups (years)					
	40–45	46–50	51–55	56–60	61–65	Total
Male	15	20	25	40	28	128
	11.72 %	15.62 %	19.53 %	31.25 %	21.88 %	
Female	13	20	36	34	39	142
	9.15 %	14.09 %	25.35 %	23.94 %	27.47 %	
Total	28	40	61	74	67	270
	10.37 %	14.81 %	22.59 %	27.42 %	24.81 %

**Table 2:** Number of polyps detected during colonoscopy for males and females

Sex	Number of polyps detected during colonoscopy					
	1 polyp	2 polyps	3 polyps	4 polyps	5 polyps	Total
Male	78	35	10	4	1	128
	60.94 %	27.34 %	7.81 %	3.13 %	0.78 %	
Female	108	25	8	0	1	142
	76.06 %	17.61 %	5.63 %	0.00 %	0.70 %	
Total	186	60	18	4	2	270
	68.89 %	22.22 %	6.67 %	1.48 %	0.74 %

**Table 3:** Number of polyps by anatomical localization

Anatomical localization in the colon and rectum	Sex		
	Male	Female	Total
Cecum	7	5	12
	3.52 %	2.67 %	
Ascending colon and hepatic flexure	25	26	51
	12.56 %	13.90 %	
Transverse colon	15	11	26
	7.54 %	5.88 %	
Descending colon and splenic flexure	12	22	34
	6.03 %	11.76 %	
Sigmoid colon and sigmorectal junction	96	81	177
	48.24 %	43.32 %	
Rectum	44	42	86
	22.11 %	22.46 %	
Total	199	187	386
	51.55 %	48.45 %

**Table 4:** Number of polyps depending on the type of polyps/Paris classification

Type of polyps/Paris classification	Size of polyps in mm				
	1–3 mm	4–6 mm	7–9 mm	> = 10	Total
Ip	1	12	13	19	45
	2.22 %	26.67 %	28.89 %	42.22 %	
Ips	9	70	14	5	98
	9.18 %	71.43 %	14.29 %	5.10 %	
Is	81	89	8	10	188
	43.09 %	47.34 %	4.26 %	5.32 %	
IIa	2	18	7	3	30
	6.67 %	60.00 %	23.33 %	10.00 %	
IIb	2	7	3	8	20
	10.00 %	35.00 %	15.00 %	40.00 %	
IIc	0	1	2	2	5
	0.00 %	20.00 %	40.00 %	40.00 %	
Total	95	197	47	47	386
	24.61 %	51.04 %	12.18 %	12.18 %

**Table 5:** Histopathological examination of the dependence of polyp type/Paris classification

Type of polyps/Paris classification	Histopathological examination				
	Hyperplastic polyps/NNC	LGD	HGD	carcinoma	Total
Ip	3	31	9	2	45
	6.67 %	68.89 %	20.00 %	4.44 %	
Ips	25	69	4	0	98
	25.51 %	70.41 %	4.08 %	0.00 %	
Is	103	80	3	2	188
	54.79 %	42.55 %	1.60 %	1.06 %	
IIa	8	21	0	1	30
	26.67 %	70.00 %	0.00 %	3.33 %	
IIb	3	12	2	3	20
	15.00 %	60.00 %	10.00 %	15.00 %	
IIc	0	1	1	3	5
	0.00 %	20.00 %	20.00 %	60.00 %	
Total	142	214	19	11	386
	36.79 %	55.44 %	4.92 %	2.85 %	

*NNC* non-neoplastic changes

During the endoscopic examination, we evaluated the polyp structure visible by standard WL imaging and then by NBI. Next, we assessed the polyp structure using optical magnification, the DF option. The structural evaluation was based on Kudo’s pit pattern classification. After WL imaging, 33.7 % of the detected polyps were potentially non-neoplastic (Kudo I, 16.32 % and Kudo II, 17.36 %); 63.2 % of the polyps were noninvasive (Kudo III_L_, 28.24 %; Kudo III_S_, 30.57 %; and Kudo IV, 4.4 %); and 3.11 % of the polyps were invasive (Kudo V). Using NBI imaging together with the DF function (NBI-DF), we detected 35.2 % potentially non-neoplastic polyps (Kudo I, 16.58 % and Kudo II, 18.65 %), 36 % noninvasive (Kudo III_L_, 28.76 %; Kudo III_S_, 28.76 %; and Kudo IV, 4.4 %), and 2.85 % invasive polyps (Kudo V) (Table 6).

**Table 6:** Kudo pattern classification of polyps observed in WL and NBI-NF

Kudo classification	Kudo pattern classification of polyps						
	Kudo I	Kudo II	Kudo III_L_	Kudo III_S_	Kudo IV	Kudo V	Total
WL	63	67	109	118	17	12	386
	16.32 %	17.36 %	28.24 %	30.57 %	4.40 %	3.11 %	
NBI-NF	64	72	17	11	111	111	386
	16.58 %	18.65 %	4.40 %	2.85 %	28.76 %	28.76 %	

We performed a comparative analysis of the polyps observed during colonoscopies and the histopathological results. Regarding the histopathological diagnosis of hyperplastic polyps in WL, we noticed a predominant majority of polyps with Kudo I (88.89 %) or Kudo II (89.55 %) pit patterns, and low-grade adenomas were primarily predicted as Kudo III_L_ (87.16 %) and Kudo III_S_ (87.29 %). High-grade dysplasia polyps (4.92 %) were described in vivo as Kudo IV (88.24 %). For malignant lesions, the mucosal pattern was usually designated as invasive pit pattern Kudo V (91.67 %) (Table 7).

**Table 7:** Correlations among polyp patterns observed during colonoscopy/WL (Kudo classification and histopathological examinations)

Kudo classification/WL	Histopathological examination				
	Hyperplastic polyps/NNC	LGD	HGD	Carcinoma	Total
Kudo I	56	7	0	0	63
	88.89 %	11.11 %	0.00 %	0.00 %	
Kudo II	60	7	0	0	67
	89.55 %	10.45 %	0.00 %	0.00 %	
Kudo III_L_	11	95	3	0	109
	10.09 %	87.16 %	2.75 %	0.00 %	
Kudo IIIs	14	103	1	0	118
	11.86 %	87.29 %	0.85 %	0.00 %	
Kudo IV	1	1	15	0	17
	5.88 %	5.88 %	88.24 %	0.00 %	
Kudo V	0	1	0	11	12
	0.00 %	8.33 %	0.00 %	91.67 %	
Total	142	214	19	11	386
	36.79 %	55.44 %	4.92 %	2.85 %	

*NNC* non-neoplastic changes

On NBI imaging with magnification, 90.63 % of the polyps designated as Kudo I were hyperplastic. Furthermore, 93.06 % of the polyps classified as Kudo II were non-neoplastic; 90.09 % of Kudo III_L_ and 92 % of Kudo III_S_ polyps were low-grade adenomas; and 94.12 % polyps with noninvasive pit patterns (Kudo IV) were high-grade adenomas. All Kudo V polyps with an invasive pit pattern had a structure of adenocarcinoma upon histopathological examination (Table 8).

**Table 8:** Correlations among polyp patterns observed during colonoscopy/NBI-NF (Kudo classification and histopathological examination)

Kudo classification/NBI-NF	Histopathological examination				
	Hyperplastic polyps/NNC	LGD	HGD	Carcinoma	Total
Kudo I	58	6	0	0	64
	90.63 %	9.38 %	0.00 %	0.00 %	
Kudo II	67	5	0	0	72
	93.06 %	6.94 %	0.00 %	0.00 %	
Kudo III_L_	8	100	3	0	111
	7.21 %	90.09 %	2.70 %	0.00 %	
Kudo III_S_	8	103	0	0	111
	7.21 %	92.79 %	0.00 %	0.00 %	
Kudo IV	1	0	16	0	17
	5.88 %	0.00 %	94.12 %	0.00 %	
Kudo V	0	0	0	11	11
	0.00 %	0.00 %	0.00 %	100.00 %	
Total	142	214	19	11	386
	36.79 %	55.44 %	4.92 %	2.85 %	

*NNC* non-neoplastic changes

We evaluated the accuracy of real-time polyp type recognition with WL imaging compared to NBI-NF imaging. We noticed no difference between the diagnostic accuracy of Kudo I and II polyps. We observed improved accuracy in the preliminary diagnoses of the Kudo III_L_ lesions (from 87.16 to 90.09 %, *p* < 0.05) and the Kudo III_S_ lesions (from 87.29 to 92.79 %, *p* < 0.01). In the case of Kudo IV pit patterns, NBI-DF increased the accuracy of preliminary diagnoses from 88.24 to 94.12 % (*p* < 0.01). Kudo V pit patterns were also more distinct on NBI-DF imaging, improving from 91.67 to 100 % (Table 9).

**Table 9:** Correlation between accuracy in polyp pattern diagnosis in WL and NBI-NFs correlated with histopathological examinations

Observation	Kudo pattern polyp classifications					
	Kudo I	Kudo II	Kudo III_L_	Kudo III_S_	Kudo IV	Kudo V
WL	88.89 %	89.55 %	87.16 %	87.29 %	88.24 %	91.67 %
NBI-NF	90.63 %	93.06 %	90.09 %	92.79 %	94.12 %	100.00 %
*p*	NS	0.06	<0.05	<0.01	<0.01	<0.05

## Discussion

A proper macroscopic evaluation of colorectal lesions is essential in determining their optimal treatments. Endoscopy with WL and pretreatment forceps biopsy is insufficient to obtain an accurate diagnosis [20]. NBI-DF is a novel, powerful tool for characterizing the mucosal surface of the large intestine because it enables the visualization of the precise microanatomies of both the microvascular and micro-surface patterns of colorectal mucosal lesions. DF is a modern diagnostic capability that, for the first time, magnifies images using natural optical methods, without losing image resolution.

The real-time prediction of the histological character of a lesion is an important function of endoscopy. Chromoendoscopy and its related Kudo classification have systematized descriptions of polypoid lesions within the large intestine. However, chromoendoscopy requires dye, a special catheter, and, most importantly, time. The ability to dye mucosal surfaces electronically with the simple push of a button would considerably simplify the use of colorization, saving the time needed for traditional dyeing. The currently recommended NICE classification distinguishes between malignant and benign lesions; however, it does not determine the histopathological type or degree of dysplasia [21].

In this study, the NBI’s magnifying observations, performed in conformance with the Kudo classification and attributing importance to the surface pattern, was compared with conventional pit pattern diagnosis using NBI without magnification. The characteristics of colorectal tumors were examined in relation to their macroscopic types. NBI-DF and NBI in real-time polyp histology prediction employed the simple polyp surface mucosal pattern classification. Using NBI-DF, mucosal surface patterns were recognized in significantly more polyps compared with NBI alone.

A two-stage optical system that utilizes WL with electronic colorization of the mucosa and magnification permitted the prediction of colorectal polyp histology with high accuracy, thereby allowing the differentiation of neoplastic polyps. Furthermore, the preliminary real-time in vivo histological assessment of colorectal polyps allowed the selection of the proper treatment technique (i.e., simple snare resection, endoscopic mucosal resection, endoscopic submucosal dissection, or surgical resection).

Another concern is the recommended method of observing polyps measuring <5 mm localized in the distal part of the large intestine. Leaving these polyps in place lowers the procedure cost; however, this choice is not cost-effective, in part because of liability concerns. Not removing the polyps is less expensive, but the sequelae of diagnosing CRC in the location of previously unremoved polyps for observation can have fatal consequences. Using our methods, all polyps can be removed. According to the PIVI criteria, we gain >90 % conformity of the macroscopic image in NBI-DF upon histopathological examination, regardless of the polyp size [22]. Nevertheless, the authors admit that this result does not sufficiently support the termination of endoscopic polypectomy.

In conclusion, this study shows that NBI with optical magnification is highly accurate in predicting polyp histology in real time using a simple pattern classification system. Characteristic pit patterns obtained by magnifying NBI endoscopy provide useful clues regarding the differentiation of adenomatous from non-adenomatous polyps in vivo, without using dye. The pit pattern observations using magnifying NBI colonoscopy were also useful for assessing the resected margins after polypectomy or endoscopic mucosal resection. It may be necessary to perform subsequent management procedures, such as hot biopsies or argon plasma coagulation procedures, when neoplastic pit patterns (Kudo’s III_L_ or IV pits) are recognized at the margins of the resected tumor. In the future, NBI might contribute to real-time histological analysis during colonoscopy, which could substantially reduce the risk of polypectomy and the costs of histological evaluation by allowing adenomatous polyps to be resected and discarded.

Used appropriately in experienced hands, this technique has potential as a valuable adjunct to standard colonoscopy in predicting the histological characteristics of colorectal polyps.
